# Mental Health Changes in Older Adults in Response to the COVID-19 Pandemic: A Longitudinal Study in Mexico

**DOI:** 10.3389/fpubh.2022.848635

**Published:** 2022-04-06

**Authors:** Diana Betancourt-Ocampo, Aldebarán Toledo-Fernández, Alejandro González-González

**Affiliations:** Faculty of Psychology, Anahuac University Mexico, Mexico, Mexico

**Keywords:** older adults, longitudinal, depression, COVID-19, mental health

## Abstract

This study examined changes in some aspects of mental health, as well as worries and behaviors related to the COVID-19 pandemic in a sample of older adults, during three different moments of the first year of the pandemic in Mexico. The sample consisted of 2,307 older adults (38% men and 62% women). The results indicated that older adults presented less concern about the COVID-19 pandemic, as well as less adherence to preventing procedures in the first wave, compared to subsequent evaluations. In addition, greater depressive symptoms, anxiety and stress were found in older adults in the last wave; however, the proportion of older adults identified with high scores in these variables was lower than that reported in national data.

## Introduction

The COVID-19 pandemic has generated a global change in terms of the health of the population, including prevention, early detection, timely and appropriate care of this disease, not only in physical aspects but also in people's mental health, partly as a consequence of the social changes to which the population of almost all countries has been subjected, characterized by social distancing, which represents a risk for the development of stress, anxiety, depression, violence and other mental health disorders (1–4).

Initially, reports indicated that the disease caused by SARS-CoV-2 affected mostly adults and that certain comorbidities were consistently associated with severe cases or even death. However, cases were later reported in other population groups, such as children under 1 year of age (5) and pregnant women (6), which complicated the global picture and opened new questions about how it was affecting various population groups, not only in physical health, but also in mental health, and not only of those who suffered from this disease, but of everyone in general.

Regarding mental health, various psychological responses to a pandemic are reported in the literature, including: maladaptation, emotional distress and defensive responses, anxiety, fear, frustration, loneliness, anger, boredom, depression, stress, avoidance behaviors, among others (which in themselves represent a health risk for individuals and communities) (2, 7, 8). Lee et al. (9) conducted a longitudinal study with people who had SARS disease in 2003, who at the time of the pandemic presented high levels of stress compared to a group of people who did not have the virus; in addition, at the follow-up assessment (1 year later), the people who had suffered from SARS not only continued to show high levels of stress, but also showed high levels of depression, anxiety, posttraumatic symptoms and psychiatric morbidity, which could be an indicator of the persistence of such symptoms, despite the time elapsed and the absence of the triggering event.

In Mexico, the first case of coronavirus was confirmed on February 28, 2020, when 85,403 cases and 2,924 deaths from COVID-19 had been reported worldwide up to that moment (10). Until then, cases had been reported in 53 countries outside of China, in six regions of America, Europe, Southeast Asia, Eastern Mediterranean, Western Pacific and Africa (11). On March 24, 2020, the Mexican Ministry of Health published the preventive measures to be implemented to mitigate and control the health risks posed by COVID-19 (12). By March 30, the General Health Council declared the epidemic of the disease generated by the SARS-CoV-2 virus (COVID-19) as a health emergency (13) and, on March 31, the extraordinary actions to address this emergency were published (14). According to these documents, the immediate suspension of non-essential activities in the public, private and social sectors, until April 30, was established aiming to mitigate the spread and transmission across the communities, to reduce overrunning of the health system and the rates of severe cases and deaths in the population residing in the national territory (15).

**Figure 1:**
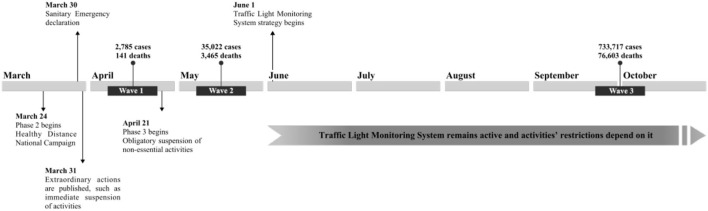
Survey timeline.

At the beginning of this study (April 7, 2020), 2,785 cases and 141 deaths due to COVID-19 were registered in Mexico (see Figure 1) (16) and, at the second recollection of data for this study (May 10, 2020), there were already 35,022 confirmed cases and 3,465 deaths due to COVID-19 (17). That is, during just 1 month there was an increase of more than 30,000 cases (~1,257%) and more than 3,000 (2,457% more). However, even with these data and with the observed trend, on May 17, 2020, the health authorities published the strategies to begin the gradual reopening of activities, including the introduction of the Epidemiological Risk Traffic Light Monitoring System (18), which would guarantee permanent monitoring of nation states regarding the regulation of the use of public space in accordance with the risk of COVID-19 contagion. The system works as follows: red light indicates that only essential economic activities can operate; orange light that, in addition to essential economic activities, companies considered as non-essential are allowed to work with 30% of their personnel, and open public spaces may operate with a reduced capacity of people; with yellow light all work activities are allowed with due precautions, and open spaces may operate regularly, though public enclosed places must operate with a reduced capacity of people; finally, green light indicates that all activities, including physical attendance to school, are allowed.

The gradual reopening of activities, called by health authorities as the “new normal” phase, began on June 1, 2020 (Figure 1), when 93,435 cases and 10,167 deaths from COVID-19 were reported (19) and when, according to the data presented by the page Our World In Data (20), there was an upward trend in both confirmed cases and deaths by COVID-19 in Mexico (21), which, at the moment of writing this report, has remained relatively constant and has only shown a considerable drop in cases on some occasions.

In addition to this, Mexico has been repeatedly questioned by international authorities regarding its control the COVID-19 pandemic; for example, being one of the countries with the lowest testing rate throughout the pandemic, estimating only 2.29 tests per thousand inhabitants by the end of May, 2020 (22), which suggests underreporting of both cases and deaths by COVID-19 in official reports by the government. Unfortunately, the different strategies implemented by the Mexican government do not seem to have had a significant impact in halting the increase in COVID-19 infections and deaths since. by the beginning of the last step of this research (September 28, 2020), the country already had 733,717 cases and 76,603 deaths (23), which placed it among the countries with the highest number of cases and deaths from COVID-19 where, according to official data, the age group between 60 and 69 years is the group with the highest number of deaths due to COVID-19 (24), despite the fact that there is a greater number of accumulated cases in people between 25 and 49 years of age (25), which confirms that older adults could be the most vulnerable to survive this disease.

It is very likely that the different moments experienced during the COVID-19 pandemic could have a psychological effect on people, especially in those population groups that are considered vulnerable, such as the elderly; however, the literature is not conclusive in this regard. For example, Di Santo et al. (26) found a reduction in healthy behaviors as a result of the preventing measures for COVID-19 (e.g., physical activity, diet, productive and cognitively stimulating activities), which could put older adults at an increased risk for dementia. In addition, the authors found a higher proportion of older adults with depression, anxiety and stress compared to data available before the start of the COVID-19 pandemic.

Li et al. (27) found that residents of regions that were quarantined due to COVID-19 had twice the prevalence of depression and anxiety compared to residents of regions where isolation measures were not applied. Ali et al. (28) conducted a study assessing the impact of COVID-19 pandemic restrictions on mental wellbeing in a sample of Bangladeshi adults, through a day-to-day comparisons of mental wellbeing measures from March 27 to April 7. They found that individuals over 50 years old showed high variability in scores compared to the younger groups of participants, suggesting greater difficulty in their mental health, according to the authors.

However, Plomecka et al. (29) assessed the mental health impact of the COVID-19 pandemic confinement on adults in 12 countries (including the USA, Spain, Italy, France, Germany, UK, Iran, Turkey and Switzerland), finding less psychological distress in older age groups, which could suggest better adaptability to this type of events. In this same sense, Röhr et al. (30) analyzed mental wellbeing in a sample of older adults during the COVID-19 closure in Germany, reporting that wellbeing in this population remained practically the same as before the closure, interpreting that, although concern about the situation was found, older adults felt socially supported and showed acceptance and resiliency in the face of these challenging conditions.

Bruine de Bruin (31), analyzing a sample of older adults in California, found greater perception of higher risks of dying if they contracted the SARS-CoV-2 virus, but with lower perceived risks of contracting the disease, being quarantined or running out of money, in addition to having low scores on depression and anxiety. Other studies (32–34) suggest that, compared to young adults, older individuals are less mentally affected by the COVID-19 pandemic.

On the other hand, García-Fernández et al. (35), found that Spanish adults over 60 years of age were less vulnerable to suffer depression and acute stress compared to younger individuals. Furthermore, they found no differences in anxiety levels during the peak of the pandemic compared to the younger group; thus, according to the authors, older people could not be considered especially vulnerable to the development of anxiety, depression and acute stress during the peak of the COVID-19 pandemic. Likewise, the results showed no gender differences for any of the clinical variables.

Stanton et al. (36) examined the associations between psychological distress and changes in healthy behaviors in a sample of Australian adults during the COVID-19 pandemic, comparing different age groups (18–45, 46–65, and over 65 years), They found that the most affected in depression, anxiety and stress were the youngest (18–45 years), and that those over 65 years showed the lower scores. However, the authors did find associations between changes in physical activity, sleep, tobacco and alcohol consumption, and severity of depression, anxiety and distress in all age groups.

As we have been able to observe, the health emergency being experienced worldwide due to COVID-19 will leave many consequences in the different countries that have been affected, but it is most likely that countries like Mexico will have a greater challenge in order to survive this crisis, due to the fact that the country already had deficiencies in health, economy and social development. Among some factors are the high prevalence of diabetes and cardiovascular diseases, job insecurity, problems of access to water and overcrowding that prevent the generalized adoption of preventive measures and multiple gaps in access to social rights, especially in vulnerable populations such as older adults (37).

In addition to this, the vast majority of the studies that have been carried out on the psychological consequences of the COVID-19 pandemic are cross-sectional, which, although valuable because they help to understand a phenomenon at a specific time, they do not allow us to know if changes can occur in the same population at different times and under different conditions, which can favor the understanding of phenomena such as the one being experienced with the COVID-19 pandemic. Thus, the present investigation examined changes over three difference timepoints in some aspects of mental health, as well as worries and behaviors related to the COVID-19 pandemic, in a sample of Mexican older adults.

## Materials and Methods

### Study Design

This is a longitudinal study, for which the Direction for the Welfare of Older Adults of the Government of the State of Mexico, provided support for the application of the instruments *via* telephone. As shown in Figure 1, the study included an initial evaluation and two follow-up evaluations: the first evaluation (wave 1) was conducted between April 7 and 17, 2020, with a total of 2,867 older adults. The second evaluation (wave 2) was completed the same year between May 10 and 22, obtaining information from 2,602 older adults who were previously evaluated. The third evaluation (wave 3) was carried out between September 28 and October 5, obtaining responses from 2,307 older adults evaluated in the two past moments; that is, the sample of this study corresponds to the same group of older adults who were evaluated at three different times.

### Data Collection and Participants

The universe of the study was older adults from the State of Mexico. According to data from the State Population Council in the State of Mexico for 2019, there was an estimate of 1,517,425 individuals aged 60 years or more. Considering a confidence level of 95% and a margin of error of 2%, the desired sample size was set at 2,398 participants.

The sample was selected non-probabilistically through a database available to the Direction for the Welfare of Older Adults of the State of Mexico. Information was obtained from the three waves of 2,307 older adults, residents from 19 regions of the State of Mexico. From the first wave, 38% of participants were men and 62% women, aged 60–100 years old (*M* = 70.69, *SD* = 7.35). As shown in Table 1, more than half of the participants indicated primary school as their highest level of education; and a high percentage indicated being married. Regarding occupation, the highest proportion of participants reported doing household chores and a second large proportion of them reported being unemployed. More than a third of the participants indicated having at least one of the following conditions: hypertension, diabetes, cancer, respiratory and autoimmune diseases, obesity, or dyslipidemia. A similar percentage reported not having any of the evaluated conditions, and approximately one fifth indicated having two or more conditions (see Table 1).

**Table 1:** Distribution of participants by sociodemographic variables.

	***N** **=*** **2,307**	
	**%**	***f***
**Sex**
Male	38.0	877
Female	62.0	1,430
**Level of education**
No schooling	15.0	346
Primary school	61.2	1,411
Secondary school	14.1	325
High school	2.9	68
Techincal career	3.5	80
Bachelor's degree	3.2	74
Postgraduate degree	0.1	3
**Occupation**
Unemployed	17.1	394
Retired	13.9	320
Employed	11.9	274
Housework	57.2	1,319
**Marital status**
Single	6.5	149
Married	66.8	1,541
Civil union	2.0	47
Divorced/separated	4.6	107
Widowed	20.1	463
**Number of people they live with**
Live alone	4.7	109
Live with someone else	28.7	662
Live with two or three people	36.5	841
Live with four or more people	30.1	695
**Medical conditions**
None	39.1	893
One	39.9	911
Two or more	21.0	480

Personnel from the Direction for the Welfare of Older Adults, consisting of nurses, social workers, psychologists and doctors, were trained for the instruments' application *via* telephone. Older adults were informed about the objective of the study, were inquired concerning their understanding of their participation, and asked to state if they agreed to participate in the study. Older adults who agreed to participate in the study were asked a couple of questions (“What date is today?”, or “Where are you right now; for example, in which town?”) to assess their sense of space and time aiming to provide evidence of sufficient cognitive capacity. In addition, the criteria and experience of the interviewer were considered (because they are professionals who work with this population everyday), to determine if the older adults were answering consistently each of the questions. The interviewers were instructed that they could interrupt the interview if they considered that there were inconsistencies or incongruities in the responses of participants.

### Measures

A sociodemographic questionnaire was used, including questions regarding sex, age, level of education, marital status, occupation, number of people the older adult lives with, and diagnosed medical conditions (hypertension, diabetes, cancer, respiratory diseases, autoimmune disease or immunosuppression, obesity, dyslipidemia).

The Impact of Event Scale-6 (IES-6), a brief form of the widely used Impact of Event Scale-revised (38), was used to assess posttraumatic stress reactions. The IES-6 includes two items for each of the dimensions of posttraumatic stress: intrusion, avoidance, and hyperarousal, and five response options ranging from 0 (“Not at all”) to 4 (“Extremely”). A total score is computed, with higher scores indicating more severe event-related stress (39). For this study, we used corresponding Spanish translated items (40), and we instructed the respondents to answer them, considering the COVID-19 pandemic as the potentially stressful event, as follows: “Some people often experience difficulties during stressful events. In the following statements, think about the last 7 days and how stressful the situation we are living due to the coronavirus pandemic has been for you.”

The Patient Health Questionnaire (PHQ-9) was also used, which is a self-administered 9-item scale that inquires the respondent about specific depressive symptoms corresponding to DSM-IV criteria in the last 2 weeks, according to a Likert scale with 0-to-3 values. A total score is computed, with upper values indicating a higher severity of depression, which can be ranked as follows: 0–4 = none, 5–9 = mild, 10–14 = moderate, 15–19 = moderately severe, 20–27 = severe (41). The PHQ-9 is a very common scale in clinical research worldwide; evidence of its validity has been reported for Latin American and Mexican populations (42, 43) and it has been used in online surveys (44, 45).

We also employed the Generalized Anxiety Disorder-7 (GAD-7), a seven-item self-administered scale based on the DSM-IV symptom criteria for general anxiety disorder (46) which also can be used to evaluate other forms of anxiety (47). Like the PHQ-9, the GAD-7 has 0-to-3 response options, with a total score between 0 and 21. Higher scores indicate higher severity of anxiety, with the following ordinal values: 0–4 = minimal, 5–9 = mild, 10–14 = moderate, 15–21 = severe. Values of good internal consistency and validity have been reported for Mexican samples (48, 49). This scale has been used in an online survey (50).

Finally, a questionnaire of concerns and behaviors related to the COVID-19 pandemic was applied, consisting of 12 items designed aster a questionnaire by Wang et al. (51). The questionnaire is divided into five areas: (a) monitoring and usefulness of prevention measures against COVID-19; (b) information about the pandemic; (c) concern about COVID-19; (d) daily-life impairments due to COVID-19, and (e) family care. The first area contains four items that evaluate how much older adults have followed hygiene and social distancing measures to prevent infection by COVID-19, as well as the usefulness that these prevention measures have. The second area contains two items that assess both how often older adults seek information about the course of the pandemic, as well as the trust they have on that information. The area of concern about COVID-19 contains four items that assess the concern that older adults feel about themselves or a member of their family becoming infected and about their economic situation and security issues in their communities as a result of the pandemic. The perception of impact by COVID-19 is assessed with an item that asks older adults how much their daily lives have been affected by the pandemic. The last area of this questionnaire assesses the perception of the elderly regarding the care provided by their family from the start of the COVID-19 contingency. Participants were instructed to respond to each of these questions in a 0-to10 scale. The granulation of the scale was established in this fashion in order to allow for a more continuous variability of the responses, for each item was considered independently in the main analyses.

### Information Analysis

Descriptive statistics were calculated for the sociodemographic characteristics of the participants. Considering the cut-off points of each of the scales used for the detection of older adults with stress, anxiety and depression, the participants were classified in each of the waves, and Cochran's Q test was performed to compare the proportions in the three waves. In addition, the total scores of the psychometric instruments, as well as each of the indicators of the questionnaire of concerns and behaviors related to the COVID-19 pandemic, were used to analyze the longitudinal changes through analysis of variance (ANOVA) for repeated measures, setting a value for statistical significance at *p* < 0.05 (two-tailed). For the effect size, η^2^ was used, considering a value equal or below 0.01 as a meaningful effect. Statistical analyses were performed with SPSS version 25 software.

### Ethical Considerations

All principles from the Declaration of Helsinki were followed. Participants were provided with information regarding the objectives of the study, the subject-matter addressed by the questions, voluntariness, and confidentiality of participation, and the institutions involved in the implementation of the study, and all were asked to provide informed consent. Before beginning the study, ethical approval was obtained from the Research Bioethics Committee from the Health Sciences Faculty, Anahuac University under the registration code CONBIOETICA-15-CEI-004-20160729.

## Results

Table 2 shows that significant differences were found in all indicators. Regarding the monitoring and usefulness of preventing measures against COVID-19, significant differences were found between the first wave with respect to the two follow-ups; that is, older adults in the first wave reported perceiving less usefulness from preventing measures, and complied less with the measures of both hygiene and social distancing compared with the two subsequent waves. Furthermore, concerning the indicators on monitoring and perceived utility of social distancing measures, a significant decrease was identified from the second to the third wave.

**Table 2:** Comparisons of means of the indicators on concerns and behaviors related to the COVID-19 pandemic by wave.

**Area**	**Indicator**	**Time**	**Comparison of means**						***Post-hoc***
			**Wave 1 (M1)**		**Wave 2 (M2)**		**Wave 3 (M3)**		
			***M***	***SD***	***M***	***SD***	***M***	***SD***	
Monitoring and usefulness of prevention measures against COVID-19	1. Monitoring of hygiene measures	*F*_(2, 2, 302)_ = 65.73, *p* < 0.001; η^2^ = 0.02	8.68	1.64	9.04	1.52	8.98	1.43	M1 < M2, M3 M2 = M3
	2. Usefulness of hygiene measures	*F*_(2, 2, 303)_ = 20.99, *p < 0*.001; η^2^ = 0.01	8.92	1.49	9.12	1.44	9.06	1.38	M1 < M2, M3 M2 = M3
	3. Monitoring of social distancing measures	*F*_(2, 2, 303)_ = 63.43, *p < 0*.001; η^2^ = 0.05	8.52	1.83	8.92	1.63	8.76	1.60	M1 < M2, M3 M2>M3
	4. Usefulness of social distancing measures	*F*_(2, 2, 303)_ = 32.15, *p < 0*.001; η^2^ = 0.02	8.80	1.60	9.08	1.53	8.96	1.46	M1 < M2, M3 M2>M3
Information on the pandemic	5. Information search	*F*_(2, 2, 303)_ = 16.42, *p* < 0.001; η^2^ = 0.01	7.55	2.41	7.83	2.32	7.72	2.32	M1 < M2, M3 M2 = M3
	6. Trust in information	*F*_(2, 2, 303)_ = 49.80, *p* < 0.001; η^2^ = 0.04	7.37	2.35	7.80	2.25	7.89	2.09	M1 < M2, M3 M2 = M3
Concern about COVID-19	7. Concern about getting infected	*F*_(2, 2, 303)_ = 61.48, *p* < 0.001; η^2^ = 0.05	8.21	2.28	8.68	2.07	8.72	1.88	M1 < M2, M3 M2 = M3
	8. Concern that a family member will get infected	*F*_(2, 2, 346)_ = 40.01, *p* < 0.001 η^2^ = 0.03	8.65	2.00	8.99	1.93	9.01	1.61	M1 < M2, M3 M2 = M3
	9. Concern about their economic situation	*F*_(2, 2, 302)_ = 23.44, *p* < 0.001; η^2^ = 0.02	8.91	1.71	9.12	1.60	8.91	1.66	M2>M1, M3 M1 = M3
	10. Concern for safety	*F*_(2, 2, 302)_ = 26.10, *p* < 0.001; η^2^ = 0.02	8.27	2.09	8.58	2.02	8.52	1.89	M1 < M2, M3 M2 = M3
Daily-life impairment	11. Impact on their daily life because of the pandemic	*F*_(2, 2, 302)_ = 116.54, *p* < 0.001; η^2^ = 0.09	8.08	2.27	8.80	1.94	8.71	1.77	M1 < M2, M3 M2 = M3
Family care	12. Family care as of the pandemic	*F*_(2, 2, 302)_ = 43.37, *p* < 0.001; η^2^ = 0.04	8.85	1.82	9.17	1.63	9.07	1.49	M1 < M2, M3 M2>M3

Older adults also reported searching less information, as well as lesser trust in the information about the pandemic in the first wave compared to the next two follow-ups; between the second and third waves, no significant differences were identified. Further, significant differences were found between the first wave compared to the follow ups. Specifically, at baseline, older adults reported having a lower concern of infection, both for them and their families, as well as a lower concern for security aspects in their community as a result of the pandemic. Likewise, the results showed that older adults reported lower impact of the pandemic on their daily life in the first evaluation than in the two subsequent waves, between which no significant differences were found. No significant difference was observed in this regard between the second and third wave. Results also showed that older adults reported greater concern in the second wave, compared to the first and third waves. Finally, results of the questionnaire indicated significant differences between the three waves in relation to perceived family care; specifically, perceived care was higher in the second wave compared for the first one, though the score dropped again in the third wave (see Table 2).

**Figure 2:**
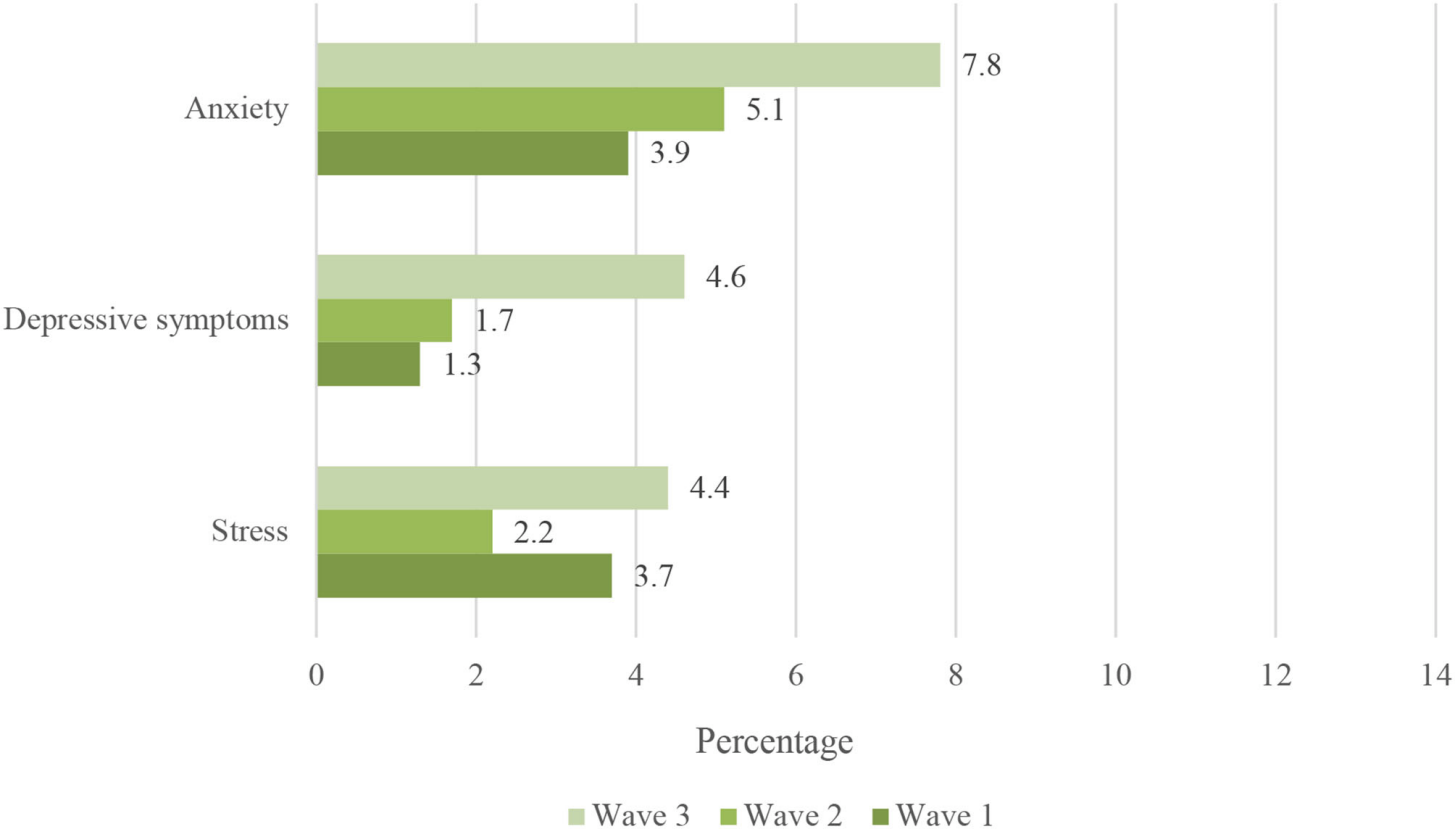
Comparison of the proportion of older adults with detection of anxiety, depression and stress in the three waves.

Regarding the differences in the proportions of older adults who presented stress, a high symptomatology of depression and anxiety was found in the three waves. Results in Figure 2 showed differences in the distributions of the proportions so much in stress [Q (2) = 20.89, *p* < 0.001], as in depressive symptoms [Q (2) = 74.14, *p* < 0.001], as well as in anxiety [Q (2) = 45.25, *p* < 0.001]. In the case of anxiety and depressive symptoms, the proportion of older adults who were classified with the diagnosis increased in each of the waves; however, for post-traumatic stress, the percentage decreased in the second wave but increased again in the third one.

When analyzing if there were significant differences in stress, depressive and anxiety symptoms throughout the three waves, the findings showed significant differences in all the variables (see Table 3). The results indicated that older adults presented higher scores in the third wave with respect to the two previous ones. Likewise, significant differences were found between the first and second wave, except for the stress variable.

**Table 3:** Comparisons of means in stress, depressive symptoms and anxiety by wave.

	**Statistical difference**	**Comparison of Means**						***Post-hoc***
		**Wave 1 (M1)**		**Wave 2 (M2)**		**Wave 3 (M3)**		
		***M***	***SD***	***M***	***SD***	***M***	***SD***	
Stress	*F*_(2, 2, 303)_ = 18.51, *p* < 0.001; η^2^ = 0.01	4.10	3.88	3.99	3.69	4.54	4.30	M3>M2, M1 M1 = M2
Depressive symptoms	*F*_(2, 2, 301)_ = 67.05, *p* < 0.001; η^2^ = 0.05	2.51	3.49	2.70	3.77	3.63	4.85	M3>M2, M1 M2>M1
Anxiety	*F*_(2, 2, 303)_ = 28.34, *p* < 0.001; η^2^ = 0.02	2.56	3.19	2.75	3.49	3.17	3.97	M3>M2, M1 M2>M1

## Discussion

The present study analyzed changes over time in behaviors and concerns regarding the COVID-19 pandemic, as well as changes in symptoms of depression, anxiety and stress at different times during the COVID-19 pandemic in a group of older adults. The findings showed that, overall, older adults reported lower scores of concerns about COVID-19 in the first wave than in subsequent waves. Although significant differences were not found in all cases between the second and third waves, the data showed a general increase in scores in the second one, which then dropped again by the last wave; however, while the scores dropped, they did not go back to the scores of the first moment. These results could be due to the moment that was being experienced, since there was a substantial increase in cases and deaths due to COVID-19 in the country (17) during the second wave. That is, the second wave was carried out at a time characterized by a significant increase in the number of cases and deaths; in addition, between 2 and 3 weeks had passed in within the third phase of the pandemic, where non-essential activities had been suspended in the country. By the third wave of this study (where scores decreased again), almost 4 months had passed since the start of the “new normal” phase, and although there were already a significant number of cases and deaths from COVID-19 (23), it appears that older adults progressively habituated themselves to an environment characterized by illness and death.

Regarding symptoms of depression, anxiety and stress, older adults presented the highest scores in the third wave, compared to the previous evaluations; however, in the case of stress, no significant differences were found between the first and second waves. In this sense, the comparison analyses between the proportion of adults who could be classified as having high scores for stress, anxiety and depression, also showed significant differences, where the proportion of older adults identified with significant symptomatology (according to the scales' cut-off scores) was higher within the third wave.

It is important to note that the percentages found were lower than those reported in national surveys, at least for depressive symptomatology, since this is the one for which information is available at a national level in this population. For example, the National Institute for Older Adults (52) suggests that depression is the most frequent affective disorder in people over 60 years of age in Mexico, and that it is present in 15–20% of the older adult population. Similarly, the National Health and Aging Survey 2018 (ENASEM), reports that 30.6% of the population aged 50 years or older, report five or more depressive symptoms, with women being the ones reporting more of these types of symptoms (37.2%) in contrast to men (21.9%) (53). In the present study, the highest percentage of older adults who were classified with high scores in depressive symptoms were 4.6% (data from the third wave), which is a percentage far below from what is reported at a national level, although we should not lose sight of the fact that the severity of depressive symptoms tends to worsen over time and that, most likely, we are only witnessing the natural course followed by this condition, given the situation that was experienced, without it having reached significant levels yet.

In addition to this, it is interesting that, although the evidence pointed to an alarming situation, mainly in terms of the number of cases and deaths, that would justify the presence of certain levels of stress, anxiety and depressive symptoms as part of an adaptive process to the prevailing situation, the narrative imposed by the federal authorities presented a different reality; that is, the messages issued indicated that the pandemic was under control, and that in a short time the situation would return to normal, which generated a sense of certain tranquility and relaxation on the part of the population.

As previously mentioned, it is very likely that the COVID-19 pandemic will have a psychological effect on people; however, by the time this paper was prepared, the evidence is still not entirely clear about the type of effects that could occur, and which populations are most affected. Some research (26–28) suggests that older adults showed some impairment in variables related to mental health, such as anxiety, depression and stress as a consequence of the COVID-19 pandemic. The present study adds to this direction of findings.

However, although effects on the mental health of the elderly were found in this study, these were of very mild effect size. It is important to consider some characteristics of this population, which apparently has been able to adapt to the changes and challenges posed by the pandemic and the social distancing, since in some way this population did not see their daily dynamics significantly altered, given that most of them were already retired, had at least one support network (mostly a spouse or partner), maintained a certain economic stability, either because of the pension they receive or because of the support received through one of the government welfare programs, which places them in a stable situation, at least in basic needs. This is consistent with a report by Röhr et al. (30), where no changes in mental wellbeing related to the pandemic were found in a sample of older adults and, although some concern about the situation was reported by these participants, they also reported high perceived social support which could have allow them to cope with the adverse conditions of the pandemic.

Concerning limitations of this study, it is important to highlight that our recruitment was non-probabilistic, and thus generalization of results is limited. The participants were older adults included in a welfare program by the government of the State of Mexico, and thus are characterized by low income and, most likely given the demographics of this region of the country, residing in places with medium-to-low conditions of living, suggesting at least some homogeneous characteristics. A second important limitation is the fact that the interviews were conducted *via* telephone, and thus responses could have been biased from both the part of the respondent and the interviewer, since non-verbal information was not available. Besides, returning to the problem of sample representation, it is likely that we excluded an important sector of the population that has no access to a telephone line. We tried to control possible interviewer bias by providing training on the administration of the questionnaires.

Despite the fact that a significant number of studies have been generated for the understanding of the mental health repercussions that the COVID-19 pandemic has had on different populations, including the elderly, we cannot ignore the fact that there are many variables that could be contributing to the way in which the subjects are facing this problem, among them those of the context itself, like the type of decisions made by the different health authorities of the countries to face the pandemic. Thus, the present research provides evidence regarding the changes in the mental health of Mexican older adults, contributing to the monitoring of mental health during this pandemic, particularly in vulnerable populations, and also adding to the international evidence to be used in cross-cultural comparative studies.

## Data Availability Statement

The raw data supporting the conclusions of this article will be made available by the authors, without undue reservation.

## Ethics Statement

The studies involving human participants were reviewed and approved by the Research Bioethics Committee from the Health Sciences Faculty, Anahuac University, Number 202003 under the registration CONBIOETICA-15-CEI-004-20160729. The Ethics Committee waived the requirement of written informed consent for participation.

## Author Contributions

DB-O established cooperation with the recruitment site. DB-O and AG-G designed the online survey and prepared the database. AT-F drafted the manuscript and conducted statistical analyses. AG-G, AT-F, and DB-O provided critical review of the manuscript. All authors participated in the conceptualization and conduction of the study. The final version of this manuscript was approved by all authors.

## Conflict of Interest

The authors declare that the research was conducted in the absence of any commercial or financial relationships that could be construed as a potential conflict of interest.

## Publisher's Note

All claims expressed in this article are solely those of the authors and do not necessarily represent those of their affiliated organizations, or those of the publisher, the editors and the reviewers. Any product that may be evaluated in this article, or claim that may be made by its manufacturer, is not guaranteed or endorsed by the publisher.
